# Identification and Mitigation of Transient Phenomena That Complicate the Characterization of Halide Perovskite Photodetectors

**DOI:** 10.1021/acsaem.2c03453

**Published:** 2023-04-17

**Authors:** Oliver D. I. Moseley, Bart Roose, Szymon J. Zelewski, Samuel D. Stranks

**Affiliations:** † Cavendish Laboratory, University of Cambridge, JJ Thomson Avenue, Cambridge CB3 0HE, U.K.; ‡ Department of Chemical Engineering & Biotechnology, University of Cambridge, Philippa Fawcett Drive, Cambridge CB3 0AS, U.K.

**Keywords:** perovskite photodetector, defects, transient behavior, photoconductive gain, photodetector characterization, passivation

## Abstract

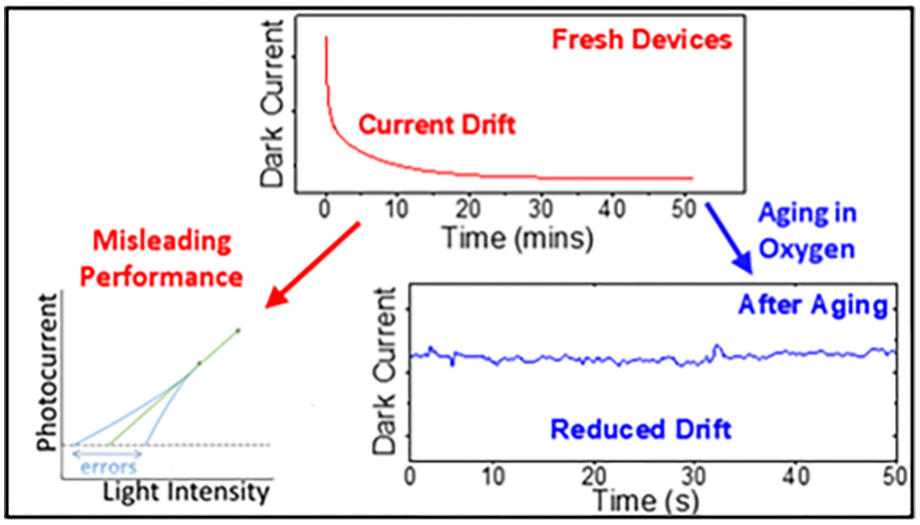

Halide perovskites have shown promise to advance the field of light detection in next-generation photodetectors, offering performance and functionality beyond what is currently possible with traditional inorganic semiconductors. Despite a relatively high density of defects in perovskite thin films, long carrier diffusion lengths and lifetimes suggest that many defects are benign. However, perovskite photodetectors show detection behavior that varies with time, creating inconsistent device performance and difficulties in accurate characterization. Here, we link the changing behavior to mobile defects that migrate through perovskites, leading to detector currents that drift on the time scale of seconds. These effects not only complicate reproducible device performance but also introduce characterization challenges. We demonstrate that such transient phenomena generate measurement artifacts that mean the value of specific detectivity measured can vary by up to 2 orders of magnitude even in the same device. The presence of defects can lead to photoconductive gain in photodetectors, and we show batch-to-batch processing variations in perovskite devices gives varying degrees of charge carrier injection and photocurrent amplification under low light intensities. We utilize the passivating effect of aging to reduce the impact of defects, minimizing current drifts and eliminating the gain. This work highlights the potential issues arising from mobile defects, which lead to inconsistent photodetector operation, and identifies the potential for defects to tune photodetection behavior in perovskite photodetectors.

## Introduction

Photodetectors sense light by converting photons into a measurable signal. Semiconductor-based detectors generate an electrical response upon illumination, and traditional devices exploit inorganic materials such as Si, InSb, and InGaAs as the light absorber. While industrially mature, there are significant advantages to replacing these materials with solution-processed alternatives, including realization of tunable response bands, low fabrication costs, and compatibility with flexible substrates. Halide perovskites are a key contender for next-generation devices due to their outstanding optoelectronic properties,^1^ including large absorption coefficients, diffusion lengths, and carrier mobilities. These properties have led to the rapid rise of perovskite solar cells, currently exceeding power conversion efficiencies (PCEs) of 25%,^2^ and similar advancements have been made with photodetectors. In less than a decade of development, perovskite photodetectors can not only outperform traditional materials,^3^ with high sensitivity^4^ and fast nanosecond response times,^5^ but the unique material properties could advance the functionality of detectors beyond current levels. For example, perovskite detectors offering narrow^6−8^ and multiple spectral response bands,^9,10^ polarization sensitivity,^11−13^ and flexible devices^14−16^ have been demonstrated in recent years.

However, despite the promise, perovskite photodetectors still face key challenges that must be overcome before commercial deployment. Foremostly, perovskite detectors have shown dynamic and variable behavior, occurring over both short (seconds) and long (months) time scales, contributing to a constantly changing performance. In the short term, the response of perovskite detectors has been shown to drift with time, linked to ion migration in the material.^17−19^ The changing performance of detectors on short time scales will be catastrophic in applications, with repeat readings from the sensor yielding different values. Moreover, this fast dynamic behavior also makes characterizing the performance of a device difficult, and representing the “real” performance becomes a challenge. Longer-term variations in performance are also problematic, as consistent performance over time scales of years is required in applications, and the stability of perovskites is an ongoing challenge.^20^ The soft nature of the perovskite lattice and the dynamic behavior of the constituent ions lead to changes over these time scales, causing variation in device performance.^21^

An understanding of the variation of the transient properties of perovskite photodetectors over these time scales is currently lacking in the literature, despite this variation being a widely acknowledged feature of the material.^22^ Herein, the dynamic behavior in perovskite photodetectors is presented and discussed, highlighting links to mobile defects. This study elucidates two important phenomena in perovskite photodetectors and provides guidelines for the community on how to correctly characterize these devices. First of all, current drift in perovskite photodetectors is closely monitored, which can lead to characterization artifacts if not properly considered. These errors wrongly exaggerate photoresponse and detectivity, and a measurement method is proposed to overcome this issue. Second, the origin of photoconductive gain in certain devices is uncovered. Vastly different performance is found between devices, even with devices produced in the same batch including the same device architecture and light absorbers. A minority of devices demonstrate photoconductive gain under low light intensities, and, interestingly, we correlate the devices that show the worse initial photovoltaic performance to those demonstrating the gain. However, after storage in dry air to induce passivation of defects, devices lose both the photoconductive gain and the drifting of current, emphasizing the important influence of defects on this behavior. This work highlights the challenges caused by mobile species and defects for accurately characterizing perovskite photodetectors, and it highlights defect engineering as a lever to control device properties to meet commercial requirements.

## Results

### Defect Migration: Current Drift and Characterization Artifacts

**Figure 1:**
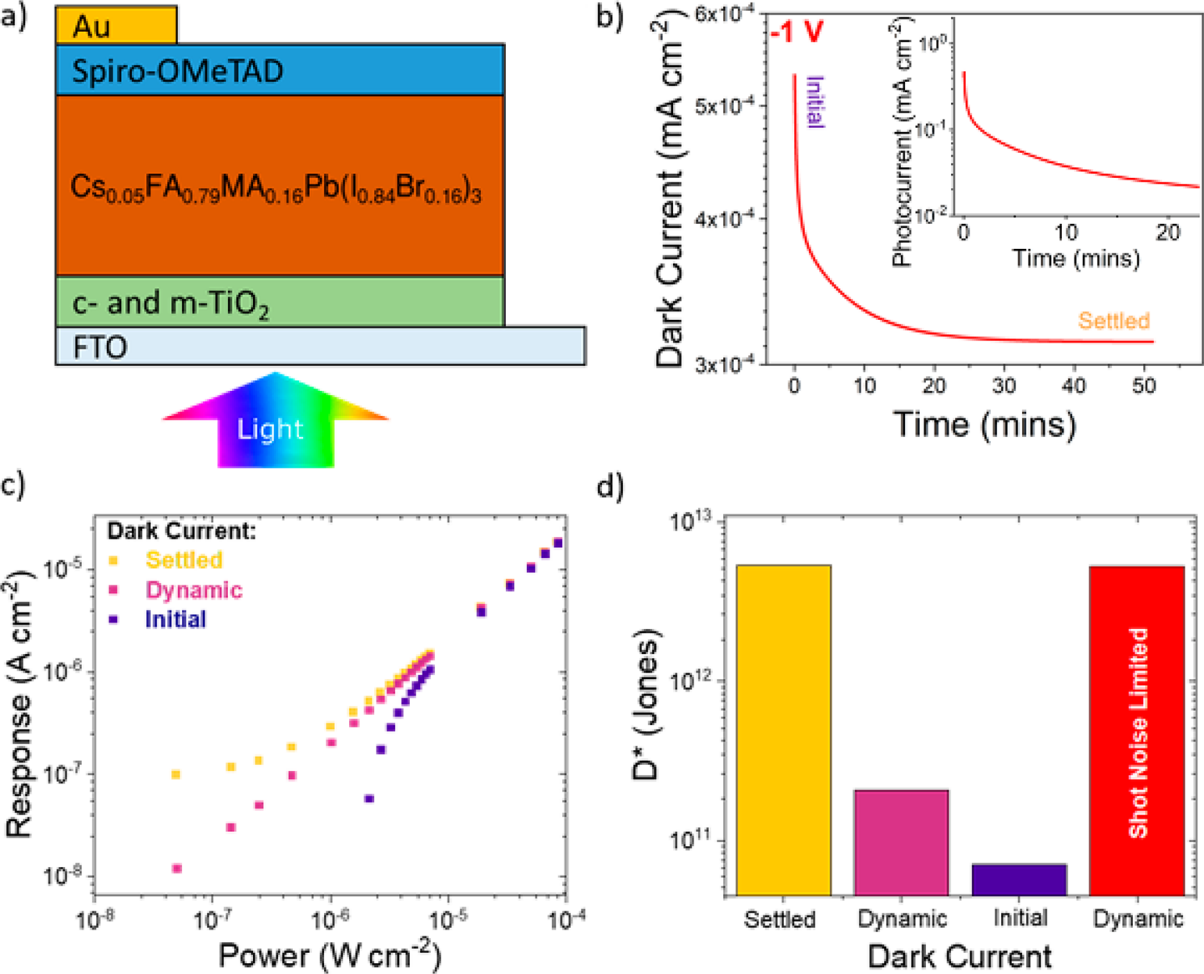
**Dark current drift and resulting characterization issues.** (a) Device architecture of the n-i-p photodiodes. (b) Drift of the dark current at −1 V, with tens of minutes needed to reach settled values. (inset) Similar dynamic behavior of the photocurrent (405 nm, 1.3 mW cm^–2^). A bias of −1 V was selected after *JV* analysis of reverse bias behavior (Figures S2 and S3). Initial and settled dark current values were used for subsequent calculations in (c, d). (c, d) The impact of different dark current values in calculating (c) the photoresponse (*I*_light_ – *I*_dark_) and (d) the corresponding detectivity, calculated using either the total noise (from a noise spectrum measurement) or assuming the system is shot noise limited (red bar). The shot noise was calculated using the average dark current obtained from the dynamic measurement.

The device structure used for this study was an n-i-p perovskite photodiode with a fluorine-doped tin oxide (FTO)/c-TiO_2_/m-TiO_2_/Cs_0.05_FA_0.79_MA_0.16_Pb(I_0.84_Br_0.16_)_3_/2,2′,7,7′-Tetrakis[*N*,*N*-di(4-methoxyphenyl)amino]-9,9′-spirobifluorene (Spiro-OMeTAD)/Au architecture (Figure 1a). We began characterization by measuring the dark current in the device. Both the dark and photocurrent vary with time under both reverse bias (Figure 1b) and short-circuit conditions (Figure S1), highlighting an example of short-term dynamic behavior. This phenomenon has been reported in the literature,^17,23^ described as current drift, and is particularly problematic to detection applications.^19,24−26^ The origins of the drift have been attributed to ionic (vacancy) migration,^18,27^ and the movement of ions opposes the applied electric field and can accumulate at interfaces to change interface energetics, causing currents to change with time.^28^ The impact on devices is a changing baseline that lowers photodetector performance, and it also introduces characterization challenges.

The characterization issues from the drifting current occur when measuring the photoresponse, *I*_response_ = *I*_light_ – *I*_dark_, required for intensity-dependent measurements (Supporting Information, Note 1). A constantly varying current means both *I*_light_ and *I*_dark_ must be measured at each light intensity. In contrast, recording just the *I*_light_, and taking a constant and independently measured *I*_dark_, brings errors into characterization. Figure 1b shows a range of dark current values could be taken for the same device and the current density–voltage (*JV*) curve could also provide an additional different dark current value (and would vary depending on scan direction, step size, and dwell time). Note that the photocurrent shows a similar dynamic behavior (inset Figure 1b).

The errors from assuming a constant *I*_dark_ will be particularly relevant at low illumination intensities, where the magnitude of *I*_light_ and *I*_dark_ become comparable. The effect of using different values of the dark current is emphasized in Figure 1c, showing the differences between using the initial (∼500 nA/cm^2^), settled (∼300 nA/cm^2^) value, or recording a unique dark current value after each corresponding photocurrent measurement (referred to as the “dynamic” measurement procedure). While a dynamic measurement leads to a linear intensity dependence down to the limits of the experimental setup, expected for a photodiode with transport layers preventing charge injection and any resulting gain, taking a fixed dark current value gives a considerably different result. Overestimations of dark current, taken from initial values in Figure 1b, lead to the appearance of sublinearity, while the opposite is the case for taking a settled dark current value. We note that the absolute dark current densities could be further lowered by employing a hole transporting material with a deeper valence band maximum, but this is beyond the scope of the current work.^29^

The observed variation in photoresponse at low illumination intensities, arising from current drifts, is problematic when assessing the ability of a photodetector to resolve weak light signals. A measure of the low-illumination detection ability is quantified by the specific detectivity (D*, see Supporting Information, Note 1), which accounts for both the photoresponse and detector noise, and the total noise spectrum of the photodetector was measured for this calculation (see Methods section for details). Subsequently, the photoresponse variation leads to a wide spread of reported values in detectivity from the same device (Figure 1d), with almost 2 orders of magnitude difference in this example here. Note that the initial and settled approaches are currently most commonly used in the field, but as can be seen in Figure 1d, this can lead to widely varying detectivity values for the same device. Using this knowledge, independent measurements of dark current were taken as standard for measurements of perovskite detectors (dynamic measurement procedure, Figure S4). Reliable measurements of detectivity require not just a truly representative photoresponse measurement but also an accurate value of the detector noise. Figure 1d also shows additional detectivity errors can arise when the noise current is incorrectly assumed to be shot noise limited, a common assumption in the literature,^30−35^ instead of a full noise spectral measurement. Again, this provides overestimations of detectivity values. Shot noise represents just a single contributing factor to the detector noise, and the actual detector noise is often higher.^4,36−39^ Therefore, the total noise from a photodetector is required to be independently measured to accurately determine the detectivity, as performed here. These points highlight the care required to correctly characterize perovskite photodetectors.

### Processing Variations and Resulting Defects Lead to Gain

**Figure 2:**
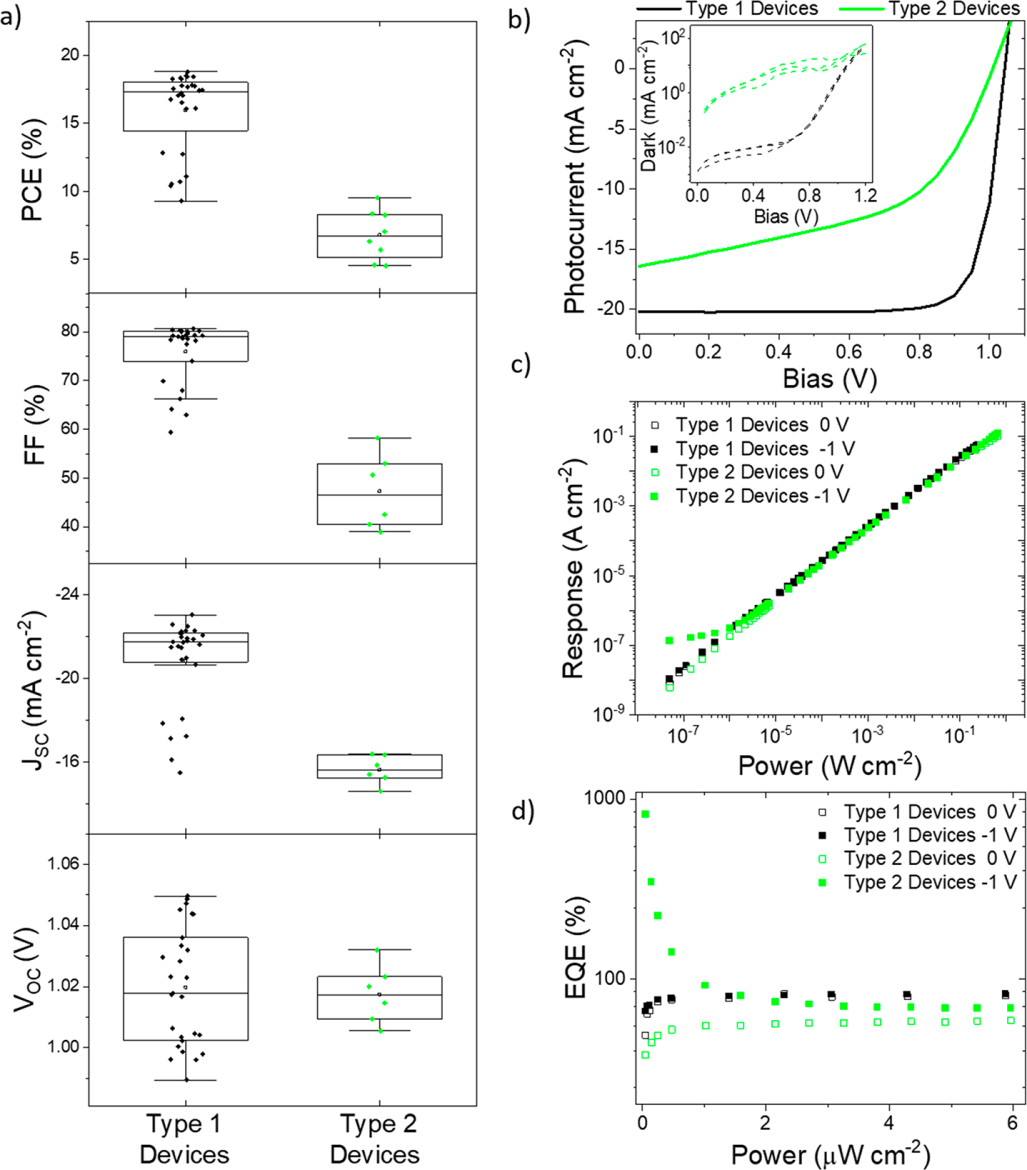
**Batch-to-batch photovoltaic and low-light photodetection performance variations.** Type 1 devices (black) and Type 2 devices (green) refer to devices showing good and bad initial photovoltaic performance, respectively. (a) Device statistics of 36 devices, categorized as either good (Type 1, black) or bad (low FF and *J*_sc_, Type 2, green) photovoltaic performance. (*V*_OC_ = open-circuit voltage, PCE = power conversion efficiency). (b) Current–Voltage curves of Type 1 and Type 2 devices under illumination (AM1.5, solid lines). Inset shows the dark current on a semilogarithmic scale, highlighting the relative difference between *R*_shunt_ values across six devices. *JV* scans were performed in the reverse direction as standard. (c) Intensity dependence of detector current response (*I*_light_ – *I*_dark_) measured at 0 V (hollow data points) and −1 V (solid data points). Measurements performed using the dynamic procedure introduced above. (d) The corresponding intensity dependence of EQE at low light intensities.

Even when employing the dynamic measurement procedure, some devices still showed photoconductive gain. To further investigate the origins of this gain, a total of 36 individual n-i-p photodiodes across two identical batches were fabricated. As an initial assessment of the device quality and to evaluate the light-harvesting ability, *JV* curves in the dark and under illumination (AM1.5) were measured. Subtle variations in processing, such as solution stoichiometry and glovebox conditions, can cause differences in performance between and within batches,^40^ and *JV* curves provide information as to what causes any differences. While the majority of devices had moderate photovoltaic performance, there were several cases of devices with lower performance, with fill factor (FF) and short-circuit current (*J*_sc_) losses. Performance can be grouped into two distinct categories with good and bad photovoltaic performance (Figure 2a), and typical examples of a “good” and “bad” device are shown in the *JV* curves in Figure 2b, referred to as Type 1 and 2 devices, respectively. The slope from 0 V to *V*_mpp_ (max power point) in Type 2 devices is characteristic of low shunt resistance, which usually arises from alternate current pathways through the device—a result of defects arising during fabrication—and leads to a reduction in fill factor.^41^ The semilogarithmic plot of the dark current (Figure 2b inset) also highlights the difference in slope near 0 V, confirming shunt pathways.^42^ The poor performing devices also suffered from *J*_sc_ losses and, combined with low fill factors, the efficiencies (PCEs) of the Type 2 devices are less than 10%. While the occurrence of Type 2 devices can be mitigated in a controlled industrial setting, it is shown below that these devices are extremely insightful to understand the underlying processes causing photoconductive gain.

The intensity dependence of Type 1 and 2 devices was measured under reverse bias and short circuit, as wider linear dynamic ranges are expected when biased (Supporting Information, Note 2), and clear differences between the two devices are seen (Figure 2c). The Type 1 devices, with superior photovoltaic performance, had a greater external quantum efficiency (EQE, 79% vs 59%) than the poorer devices without applied bias at moderate light intensities. Under reverse bias, there is negligible improvement for the Type 1 device, whereas Type 2 devices show an improvement in sensitivity. These findings can be expected, due to efficient collection of charge carriers in Type 1 devices, which do not require additional bias to facilitate carrier collection. Second, the low-light behavior of both devices is significantly different. For Type 1, devices display the expected linearity and a wide linear dynamic range (LDR) of 147 dB, while Type 2 devices deviate from linearity due to an increase in sensitivity at low light intensities (Figure 2c). This limits the LDR of Type 2 devices to 87 dB but amplifies the response under low-light conditions. Converting each photoresponse into the EQE at each incident intensity shows this superlinearity in more detail (Figure 2d), and below 1 μW cm^–2^ the EQEs begin to increase for Type 2 devices. The EQE continuously increases as light intensity decreases, exceeding 100%. This low-light behavior is repeated across several Type 2 devices, all with poor initial photovoltaic performance (Figure S5). The superlinear behavior is consistent with photoconductive gain originating from long-lived trap states to increase carrier lifetimes (see Supporting Information, Note 1).^43−45^

The absence of any photoconductive gain in Type 1 devices likely stems from the lack of charge carrier injection, preventing recirculation of charge carriers and any amplification of current beyond photogenerated contributions. This is verified by the *JV* behavior seen in Figure 2b, indicative of good diode-like behavior. In contrast, Type 2 devices demonstrate photoconductive gain at low light intensities when reverse biased, meaning injection from the electrodes into the perovskite likely occurs in order to generate this amplification of the photocurrent. Charge carrier injection is possible with direct electrode-perovskite contact through processing inconsistencies such as pinholes and voids in the device layers formed during fabrication. We propose that the charge injection could occur at the (i) bottom (perovskite-FTO) interface or at the (ii) top (perovskite-gold) interface. For (i), it has been shown elsewhere that the replacement of a compact TiO_2_ layer with a porous TiO_2_ layer reduces shunt resistance,^46,47^ and FTO-perovskite contact through TiO_2_ has caused photocurrent amplification in similar device structures.^44^ When only porous TiO_2_ was used, and direct perovskite-FTO contact is possible, photoconductive gain occurs. However, when a conformal and compact layer of TiO_2_ prevents direct contact, the amplification is lost.^44^ In our case, all devices use a compact layer of TiO_2_, which in most cases (Type 1 devices) prevents direct contact and no amplification is observed. However, pinholes in the compact TiO_2_ layer due to processing variations may allow direct contact and photoconductive gain (Type 2 devices). For (ii), thickness inconsistencies and high surface roughness in the perovskite film can lead to incomplete hole transport layer coverage and charge injection via direct perovskite-gold contact at the top device interface. Variations in perovskite thickness can also explain the reductions in *J*_sc_ in Type 2 devices. Regions with excess thickness reduce current extraction through poor collection efficiency, whereas current is lost in areas with insufficient thickness from absorption losses. Further work will be required to unambiguously identify the origins of the photocurrent amplification mechanism. Overall, these findings show that two devices of the same architecture can show significant variation in their photodetection through batch-to-batch or even device-to-device differences in their processing, emphasizing the importance of measuring down to low illumination levels to reveal these effects.

**Figure 3:**
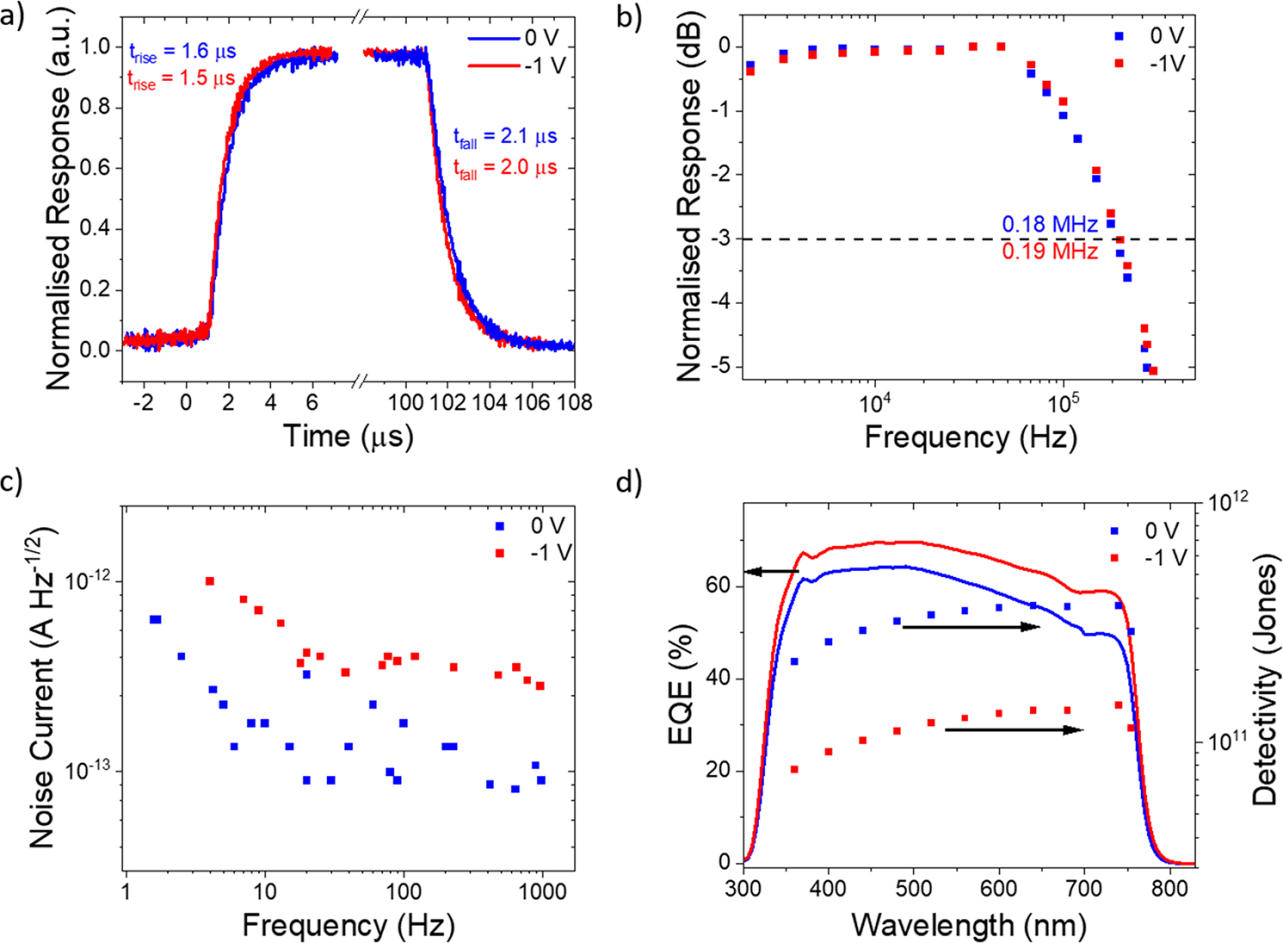
**Photodetector performance of Type 2 photodetectors under reverse bias.** Blue and red represent 0 and −1 V performance, respectively. (a, b) The response speed, with (a) showing rise and fall times and (b) the *f*_–3 dB_ cutoff frequency. (c) Noise spectral density. (d) EQE and corresponding detectivity estimations. Response speed and EQE were measured with illumination levels above the regime where gain was observed (>10 μW cm^–2^).

Further characterization of the photodetection performance of a Type 2 device was performed, with measurements repeated under reverse bias to assess the impact of photoconductive mode operation on performance. The response to pulsed light is displayed in Figure 3a,b. Rise and fall times were found to be 1.6 and 2.1 μs at no applied bias, improving slightly to 1.5 and 2.0 μs under −1 V reverse bias. Similarly, slight increases in cutoff frequencies (*f*_–3 dB_) were seen under reverse bias, with 0.18 and 0.19 MHz seen at 0 and −1 V, respectively. Note, the light intensity was above the regime where gain was observed due to limitations on the sensitivity of the temporal detection electronics, and so there was no detriment to response speed.^43^ Improvements in response speed under reverse bias are expected when devices are limited by transit times. The observation of only slight improvements in response speed after applying bias suggests that the response speed of the device is limited by the resistance-capacitance (RC) constant and the large capacitance related to the mesoporous TiO_2_ structure. The similar current densities at 0 and −1 V (Figure 2d) provide another indication that charge extraction is not limiting rise and fall times, indicating that there is no significant barrier for charge extraction at 0 V. Although state-of-the-art perovskite devices have response speeds on the order of nanoseconds,^5,48^ these are in highly optimized devices, with active areas as low as 0.04 mm^2^.^49^ Here, the device area remained larger at 12 mm^2^ (as per typical photovoltaic (PV) testing active areas),^50−52^ and response speeds would be expected to decrease further on reduction of this area, allowing fairer comparisons.

The noise density spectra show a slight bias dependence (Figure 3c). Both spectra are independent of frequency above 10 Hz but show an increase in noise at lower frequencies. Increased noise under reverse bias is expected, due to greater contributions from shot noise induced by larger dark currents. Also reverse bias has been shown to increase 1/*f* noise in organic photodiodes,^53,54^ and similar effects would not be unexpected in perovskite devices due to the common disorder in solution-processed semiconductors. The noise measurement was combined with EQE spectra (Figure 3d, light intensities above 1 μW cm^–2^) to provide estimations of the detectivity, with peak values of 1.4 × 10^11^ Jones (−1 V) and 3.7 × 10^11^ Jones (0 V). These values compare similarly to other n-i-p devices, with state-of-the-art approaching 10^12^ Jones,^37^ while commercial Si detectors show 4 × 10^12^ Jones.^43^

### Mitigating Transient Defects with Passivation through Aging

After initial measurements, Type 2 devices were stored in a low-humidity desiccator for approximately one year after the initial fabrication and analysis, and the impact of this storage was assessed by remeasuring the photodetector parameters. The method of storage was selected in order to protect the devices from moisture-induced degradation.^55^ Aging has been shown to be beneficial for devices in some cases. This has been linked to oxygen passivation,^56−60^ ion migration,^61^ and diffusion of ions from contact materials or even glass.^62^

**Figure 4:**
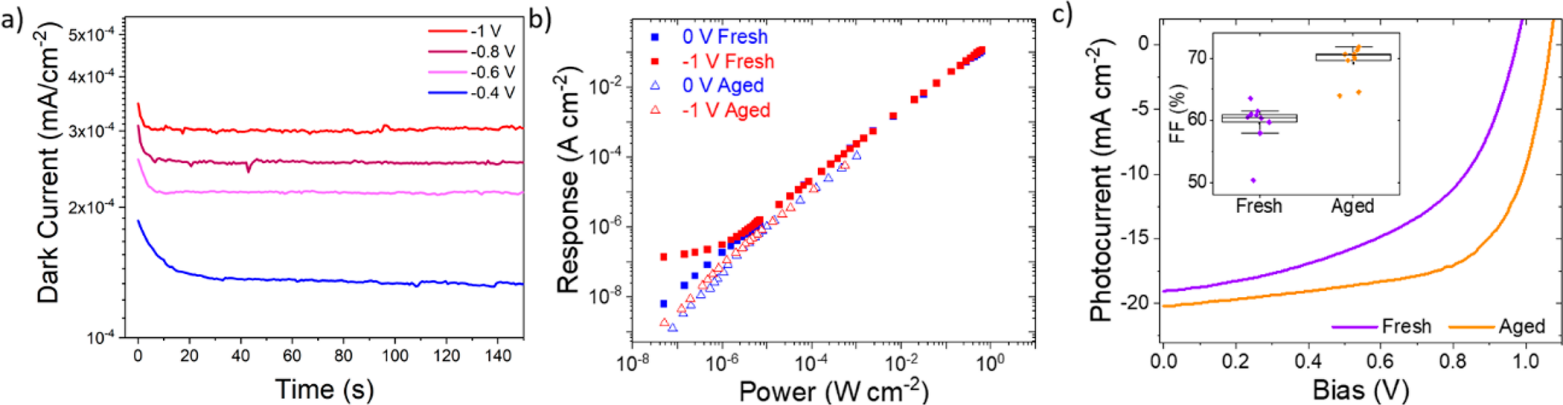
**Type 2 photodetectors after aging.** (a) Transient behavior of the dark current under reverse bias, no longer displaying the long settling times seen in fresh devices (tens of minutes, Figure 1b). (b) Intensity dependence of aged (hollow triangles) compared to fresh (solid squares) devices. (c) *JV* curves of fresh and aged devices, with the FF statistics of 9 Type 2 devices, inset.

After aging, Type 2 devices showed a significant reduction in current drifts (Figure 4a). Dark currents now reach stabilized values after several seconds, whereas before this would take hours, suggesting reduced ion migration in the aged samples. The same reduction in drift is also seen for aged Type 1 devices (Figure S6). This finding suggests that the perovskite film has been passivated during aging, reducing the density of trap states, with fewer mobile defects. It is interesting to note that, while the magnitude of the dark current depends on the applied bias, the absolute current drift is similar for all applied biases (Figure S7), indicating that the phenomenon causing drift is not significantly affected by the voltage applied.

The intensity dependence of the photoresponse in aged devices was then remeasured using the same protocols established earlier, with significant differences seen compared to the measurements on freshly made and tested Type 2 devices (Figure 4b). We note that both Figures 3d and 4b show only an ∼10% difference in current response between 0 and −1 V. Importantly, the aged devices show a clear loss in photoconductive gain at low light intensities. The absence of gain has two possible origins: removal of shunt pathways preventing charge carrier injection under bias or a reduction in the charge carrier lifetime (relative to carrier transit times). The passivation of the perovskite film after aging would be expected to increase carrier lifetimes in the low-illumination regime by reducing competing trap-assisted recombination pathways.^56,63,64^ Therefore, the removal of photocurrent amplification likely occurs due to the removal of the charge injection process.

Halide perovskites have been demonstrated to degrade and form byproducts when aged in the presence of oxygen, such as PbI_2_, PbO, and Pb^0^,^65−68^ which could act to block the direct perovskite-electrode contact. *JV* curves, measured on both fresh and aged (1 week) Type 2 devices, demonstrate a clear improvement in fill factor after oxygen aging exposure (Figure 4c) suggesting that the original shunt pathways that enabled charge injection and thus gain have been greatly reduced. Aging in the presence of oxygen has been shown to generate PbO, instead of detrimental metallic Pb^0^, which is seen when aged under vacuum, which contributes to improvements in photoluminescence in aged films.^67^ Similarly, MAPbI_3_ perovskites have been shown to degrade in oxygen, forming PbI_2_ in the reaction.^65,66^ Formation of these products at the transport layer interface would generate a barrier to charge injection, and this is supported by the susceptibility of the perovskite surfaces to aging effects.^69−72^ Continued aging results in a reduction of charge collection efficiency (Figure S8), which could be due to the insulating nature of degradation species limiting current extraction. This finding suggests that aging presents a compromise between healing the processing defects and maintaining efficient charge collection in the photodetector. Work identifying the exact cause of the gain removal in Type 2 devices passivated after aging is ongoing. Overall, this demonstrates the ability to alter the properties of perovskite photodetectors after fabrication, through aging and passivation.

## Discussion

We have highlighted the influence of defects on the performance and characterization of halide perovskite photodetectors. First, the presence of considerable defect densities in halide perovskite films introduces a requirement to measure performance under low light intensity, where the density of trap states is comparable to charge carrier densities, and their influence on performance is significant.^73^ However, defects also lead to challenges for measuring performance under low light intensities, where defect-migration-induced current drifts are of the same magnitude as the photoresponse. Therefore, care must be taken in measuring the low-light response of perovskite photodetectors. Second, the influence of defects on photodetector performance, shown here, demonstrates an additional material attribute that can act as a controllable lever on device performance. Defects and trap states contribute to generating photoconductive gain in perovskite photodetectors, by extending carrier lifetimes beyond transit times or influencing interface energetics to allow charge injection.^43,74^ Defect density, position, and trap state depth are three parameters that impact the magnitude and light intensity dependence of photocurrent amplification.^75^ By specific engineering of these defects, they can allow greater photoresponse in particular light intensity regimes and be designed to suit particular applications.

However, while defects and processing variations allow photoconductive gain to amplify response, this also leads to slower transit times and small LDRs. Additionally, as shown, defects also generate current drifts that are undesirable for commercial applications and cause characterization difficulties. Designing a fast detector with a wide linear response and stable current output can be targeted by passivation. We have used oxygen here as a lever to modulate the defect states following previous reports.^56−59^ However, its influence is hard to control, and it eventually leads to the degradation of the perovskite film and reductions in detector sensitivity (Figures 4b & S8). Therefore, other more targeted passivation approaches may be more fruitful, including but not limited to, alkali metal ions,^76^ nonstoichiometric PbI_2_,^77^ and Lewis bases.^78^ Defect migration can be specifically targeted by the removal of the grain boundary channels that facilitate their transport,^79−81^ through grain-boundary passivation^82−84^ or grain-size engineering.^79,85,86^ Reducing perovskite structural dimensionality has been used to increase the activation energies of migration^87,88^ and resultantly has found use to overcome current drifts in direct X-ray detectors, which are particularly susceptible to this effect due to the large external biases.^89−91^ The methodology outlined here will act as a platform to further test and validate new passivation approaches.

## Methods

### Device Fabrication

Chemicals were purchased from Sigma-Aldrich, unless stated otherwise.

Fluorine-doped tin oxide-coated glass was cleaned by sonication in 2% Hellmanex III solution (in deionized water) for 15 min, rinsed with deionized water, and sonication in isopropanol for 15 min. Substrates were dried and transferred to a hot plate and heated to 450 °C. Compact TiO_2_ was deposited by spray pyrolysis of a solution containing 9 mL of ethanol, 0.6 mL of titanium(IV) diisopropoxide bis(acetylacetonate), and 0.4 mL of acetylacetone. Substrates were cooled to room temperature before the mesoporous TiO_2_ (150 mg mL^–1^ paste (Greatcell Solar Materials) in ethanol) was deposited by spin coating (4000 rpm, 10 s, 2000 rpm/s ramp). After spin coating, the substrates were transferred to a hot plate preheated at 125 °C, and the following protocol was used for annealing: 10 min at 125 °C, 15 min ramp and 5 min dwell at 325 °C, 5 min ramp and 5 min dwell at 375 °C, and 5 min ramp and 30 min dwell at 450 °C. Substrates were then allowed to cool to 150 °C, after which they were transferred to a N_2_-filled glovebox for perovskite deposition. Perovskite films were deposited from a precursor solution containing FAI (1 M, Greatcell Solar Materials), PbI_2_ (1.1 M, TCI), MABr (0.2 M, Greatcell Solar Materials), PbBr_2_ (0.22 M, TCI), and CsI (0.075 M) in anhydrous dimethylformamide (DMF)/dimethyl sulfoxide (DMSO) 4:1 (v/v). The perovskite solution was spin-coated in a two-step program at 1000 and 6000 rpm for 10 and 20 s, respectively. During the second step, 100 μL of chlorobenzene was poured onto the spinning substrate 5 s prior to the end of the program. The substrates were then transferred to a hot plate preheated to 100 °C and annealed for 60 min. After cooling down to room temperature, spiro-OMeTAD (0.07 M, Lumtech) in chlorobenzene, doped with *t*-butylpyridine (3.3 mol mol^–1^), bis(trifluoromethane)sulfonamide lithium (0.5 mol mol^–1^), and tris(2-(1*H*-pyrazol-1-yl)-4-tertbutylpyridine)-cobalt(III)tris(bis(trifluoromethylsulfonyl)imide) (0.05 mol mol^–1^) was deposited by spin coating (4000 rpm, 20 s). Devices were finished by thermal evaporation of 80 nm gold, to produce a device area of 10 mm^2^.

Devices were stored long-term in a desiccator cabinet, with less than 14% relative humidity, maintained by silica beads and N_2_ gas flushing.

### Photodetector Characterization

#### JV Curves and Current Versus Time

Current density–voltage (*JV*) curve measurements were recorded with a source meter (Keithley 2636A) controlled by a custom LabVIEW program. Light measurements were performed using various illumination sources, and the wavelength and intensity specified when used. Illumination at 1-sun (100 mW cm^–2^, AM 1.5G reference air mass spectra) was provided by a xenon lamp (Abet Sun 2000 Solar Simulators, AAB class), calibrated with a reference silicon diode (KG5 filter). The voltage scan range was dependent on the application using a step size of 20 mV and a dwell time of 100 ms, and scans were performed in the reverse direction from high to low bias.

Current–Time measurements were performed using a source measure unit (Keithley 2450), which applied the stated bias voltage and recorded the corresponding device current.

#### Response Time

Transient measurements were performed using a 670 nm fiber-coupled diode laser (iFLEX). The laser beam was passed through an acousto-optic modulator (IntraAction AOM-80, ME-80 driver) to ensure fast excitation and broad frequency range, outputting 100 μW at the first order diffracted beam. The photodetector current response was recorded with a FEMTO DHPCA-100 transimpedance amplifier combined with an oscilloscope (response time measurement, Tektronix TDS2024C) or a high-speed lock-in amplifier (cutoff frequency measurement, Zurich Instruments HF2LI). The gain of the DHPCA-100 was set to 10^3^ V/A to maximize the bandwidth of the measurement. A Si reference photodiode (Thorlabs FDS010) was used to test the speed limitations of the measurement system (Figure S9). Rise and fall times were taken as the time for the signal to change from 10% to 90% of the peak response.
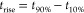


Cutoff frequency measurements take the frequency where the response falls to −3 dB of the peak response as the device bandwidth.

#### Intensity Dependence of Photoresponse

Steady-state intensity-dependent measurements were made using a 405 nm continuous wave (CW) laser (Photon Etc 405–2W). Power was reduced using neutral density filters and calibrated using an optical power meter (PM100D, Thorlabs) with a Si photodiode sensor (S130C, Thorlabs). The current was recorded using a Keithley 2450 source measurement unit (SMU), and the response was calculated by subtracting the photocurrent from a dark current measured at each intensity. Photoresponses were then converted to responsivity (*R*) or EQE using the following equations
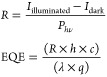
where *I*_light_ and *I*_dark_ are the light and dark photocurrents, *P* is the optical power, *h* is Planck’s constant, *c* is the speed of light, λ is the wavelength of the incident light, and *q* is the electronic charge.

Taking readings of dark current at each light intensity compensates for any current drift, which can lead to errors in calculations. The linear dynamic range (LDR), which represents the range of incident intensity for which the detector response is linear, was calculated from the following equation
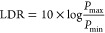
where *P*_max_ and *P*_min_ represent the upper and lower power bounds of the linear response.

#### Responsivity/EQE

EQE spectra were measured using a Bentham PVE300 system. A dual xenon short arc and quartz halogen lamp were utilized as the light source, with a swingaway mirror set to 750 nm (this is moved if it coincided with narrowband peak). A 10 × 10 mm Si reference cell was used to calibrate the power of the probe beam. Measurements in AC mode use an optical chopper, with a low-noise preamplifier and lock-in amplifier as detection electronics. DC measurements had a DC amplifier. External voltages were applied across the device using a Keithley 2450 source measurement unit (SMU).

#### Noise

Noise spectral densities were measured in the dark using an electrically shielded box and low noise current preamplifier (Stanford, SR570) connected to a lock-in amplifier (Stanford, SRS830) set to measure noise. Bias was supplied using the preamplifier.

For calculations assuming the detector noise is limited by the shot noise, the shot noise was calculated using the following equation, where *q* is the elementary charge and Δ*f* is the measurement bandwidth.
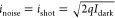


#### Detectivity Estimations

Calculating the detectivity of a photodetector requires the measurement of the noise equivalent power (NEP). Without an exact measurement of the NEP from an intensity measurement down to the noise floor, the NEP can be estimated by measuring the responsivity for a given light intensity and the device noise (*i*_noise_*)*.
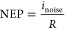


Detectivity can be estimated using this NEP value.
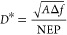


## Data Availability

The data and code that support the findings of this study are available at the University of Cambridge Apollo repository.

## Supporting Information Available

Details on the intensity dependence measurement protocols, device statistics, and aged device performance (PDF)
