# Supplementary material for: Identification and Mitigation of Transient Phenomena That Complicate the Characterization of Halide Perovskite Photodetectors
Source: ACS Appl Energy Mater. 2023 Apr 17;6(20):10233–42. doi: 10.1021/acsaem.2c03453 (PMC10598628; doi:10.1021/acsaem.2c03453)
Supplement: Supplementary file 1 — ae2c03453_si_001.pdf [file ae2c03453_si_001.pdf]

Supporting Information: Identification and mitigation of transient phenomena that complicate the characterisation of halide perovskite photodetectors

Oliver D. I. Moseley[1], Bart Roose[1,2], Szymon J. Zelewski[1], Samuel D. Stranks[1,2]*

1: Cavendish Laboratory, University of Cambridge, JJ Thomson Avenue, Cambridge CB3 0HE, UK.

2: Department of Chemical Engineering & Biotechnology, University of Cambridge, Philippa Fawcett Drive, Cambridge CB3 0AS, UK.

* Email sds65@cam.ac.uk.

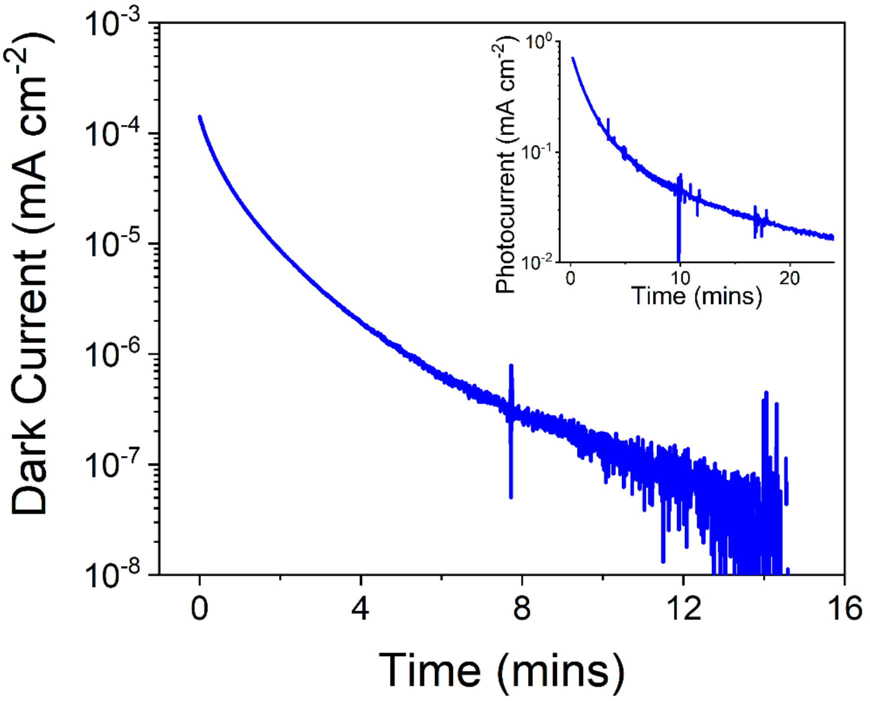

Figure S1: *Drift of the dark current under short-circuit conditions, with the corresponding photocurrent shown in the inset (405 nm, 1.3 mW cm^{-2}).*

Note 1: Photoresponse Intensity Dependence and Low Light Illumination

To characterise the photodetectors, we performed an incident light intensity dependence of photoresponse, which provides a range of figures of merit of a photodetector (Figure S3) and importantly, how these change in different illumination regimes. The linear dynamic range can be obtained from the range of incident powers between which the response is linear. The responsivity can be obtained from the gradient, and any variations in sensitivity as light

intensity changes can be determined. Finally, if the detection measurement set-up is sensitive enough, the intensity can be reduced to find the point where the response is indistinguishable from the device noise, the noise equivalent power (NEP). From the NEP the detectivity can be determined, using the following equation:

$$D^* = \frac{\sqrt{A\Delta f}}{NEP}$$

Where A is the detector area and Δf is the measurement bandwidth. This gives a wide picture of the detection performance from a photodetector, and how these change with intensity. Then the temporal response can be determined separately to give the speed of the detector.

Intensity dependence measurements are sometimes performed over a small intensity range, and then linearly extrapolated to the independently measured noise level, assuming the responsivity of the photodetector remains constant. However, this is not always the case and responsivity has been shown to vary with light intensity, leading to potential errors in LDR, NEP and D* values. For example, deviations from linear light intensity dependence measurements are commonplace in detectors exhibiting gain in their response, as introduced by Fang et al.[3] Depending on the mechanistic origin of the gain, the impact of light intensity is different, showing both super- and sub-linear behaviour.

Photodetectors based on solution processed semiconductors, possessing a high density of interfacial defect states have displayed sub-linear deviations from linear intensity dependences at low light intensity.[4–7] When the trap states at the interfaces are filled with charge carriers, band-bending can occur and charge injection through the transport layer enables photoconductive gain, increasing responsivity. However, as light intensity is reduced, and the filling of these traps no longer occurs, this gain mechanism is lost, and a reduction in responsivity is seen. Some devices only show gain at reduced light intensities, demonstrating super-linear deviations at low illumination levels.[8,9] These systems require carrier densities to

be on the order of defect densities, increasing the fraction of carriers in trap states. The trapping of one carrier (either electrons or holes) increases the lifetime of the other character, by reducing the likelihood of recombination. The increase in lifetimes of carriers relative to the carrier transit time enables re-circulation of carriers and gain. At lower light intensities, the deepest and longest lived traps are filled, enhancing gain further. Similarly, in systems without gain, but high trap densities, sublinear deviations can also be seen at low intensities where trap-assisted non-radiative losses reduce the charge carrier collection efficiency. As a result, measurements were performed down to the lower limits of our experimental set-up, in order to accurately report the figures of merit, and also investigate any trap related phenomena that may be present.

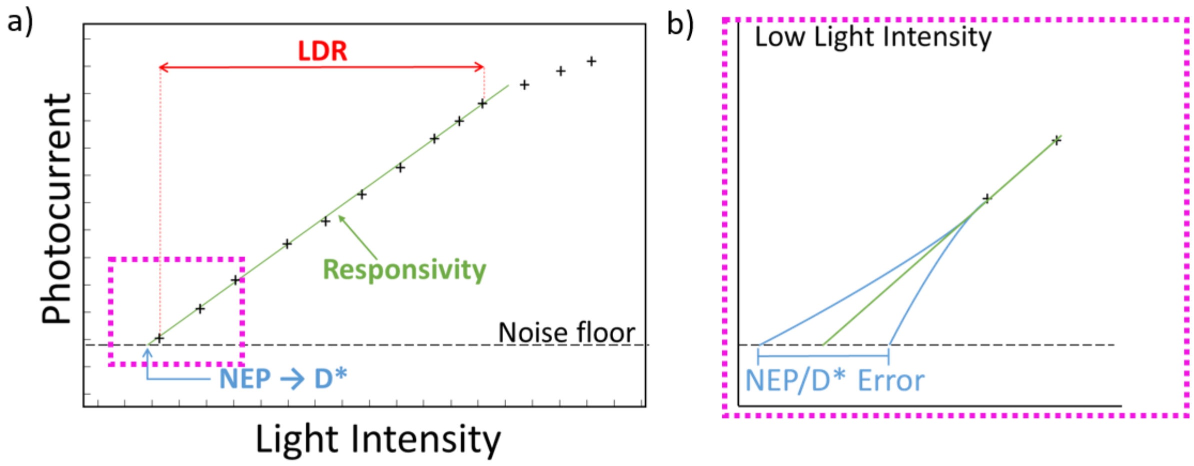

Figure S2: *a) and b) Schematics depicting the photoresponse intensity dependence of a photodetector, revealing several figures of merit. b) The low light region of the intensity dependence. This is important to measure as the response may not be linear to the noise floor, especially in detectors with high defect densities, which can lead to characterisation mistakes and missing of features such as gain.*

Note 2: Reverse Biasing

All measurements were performed with and without reverse bias, to assess any changes in photodetector performance and any additional dynamic behaviour that may arise. Photodiodes

can function without an external bias due to the in-built electric field, known as photovoltaic or self-powered mode. However, in applications they are often operated under reverse bias, known as photoconductive mode, due to enhanced perfromance.[1] Reverse bias can increase the response speed of a photodiode through two effects: increasing the drift velocity of carriers through the device, and lowering the diode capacitance by widening the depletion region. Moreover, improvements in charge dissociation and collection with an externally applied bias results in improved linearity of response and a wider LDR.[2] However, reverse bias also increases dark current, through increased charge injection, which increases the shot noise from the device. Also, excessive reverse bias can degrade the device, and so care must be taken to balance any improvements in performance.

The amount of reverse bias was selected after IV curve analysis of the devices (Figure S2), ensuring the bias was well before the breakdown of the device. Commonly, this was selected as -1 V.

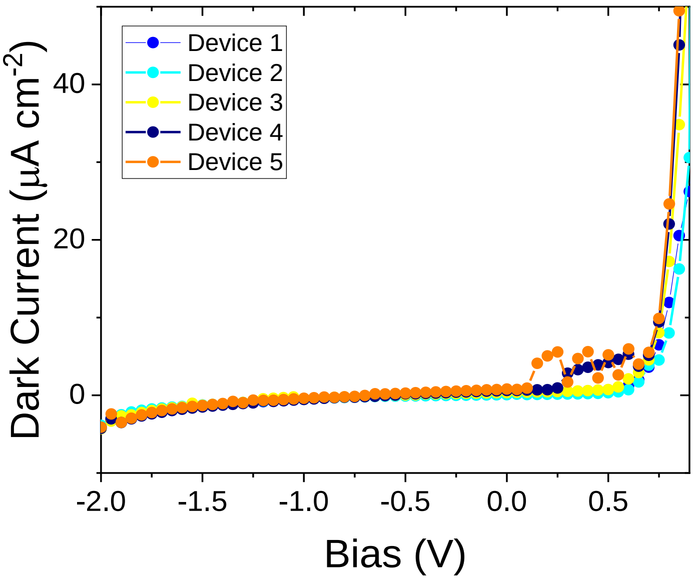

Figure S3: *Initial measurement of the dark JV curves to select a reverse bias that is below the breakdown voltage. Reverse biases of -1 V were subsequently used as standard.*

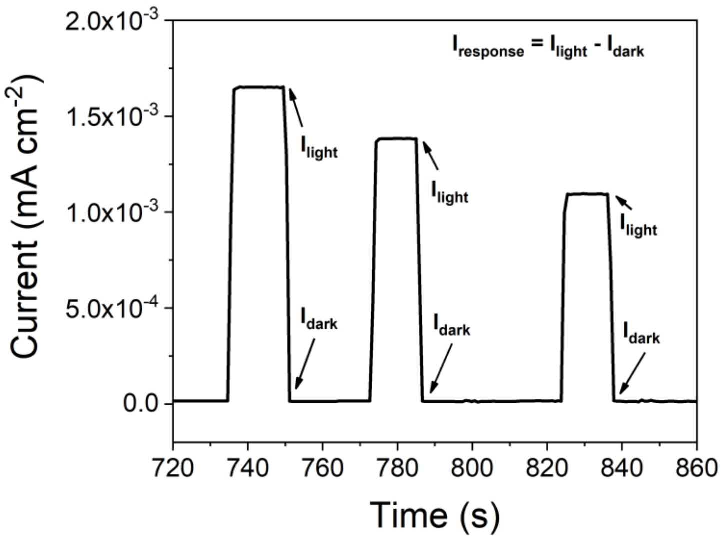

Figure S4: *Measurement protocol for intensity dependence of photoresponse, referred to as the 'dynamic' measurement procedure. Taking a measurement of I_{light} and I_{dark} at each illumination intensity avoids any characterisation issues that arise when the current drifts with time.*

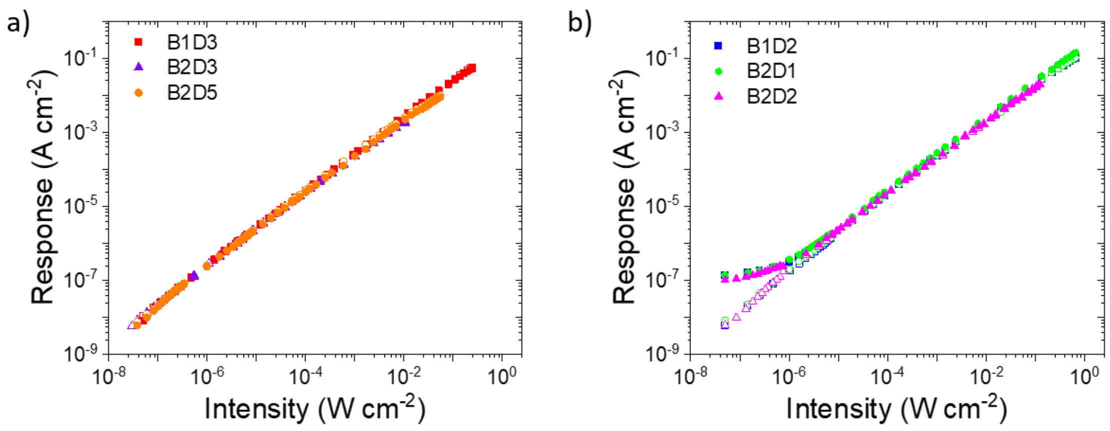

Figure S5: *Photoresponse intensity dependence for other Type 1 (a) and Type 2 (b) devices.*

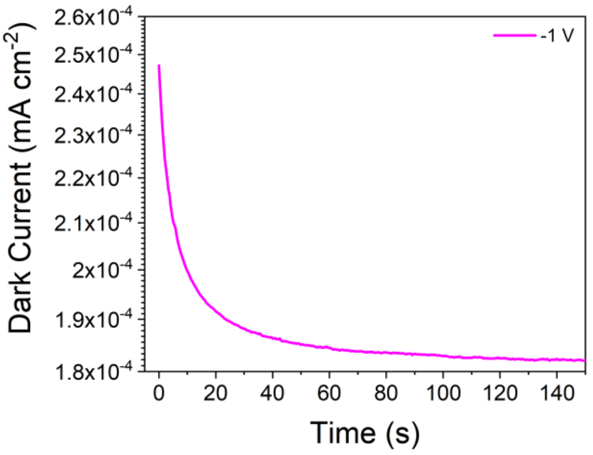

Figure S6: *Current drifts for aged Type 1 devices.*

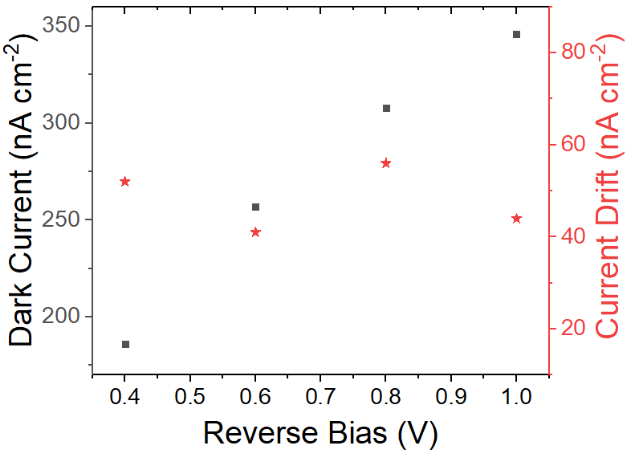

Figure S7: *Current density variation $J_{final} - J_{initial}$ vs. applied reverse bias, for the transient current curves in Figure 4a*

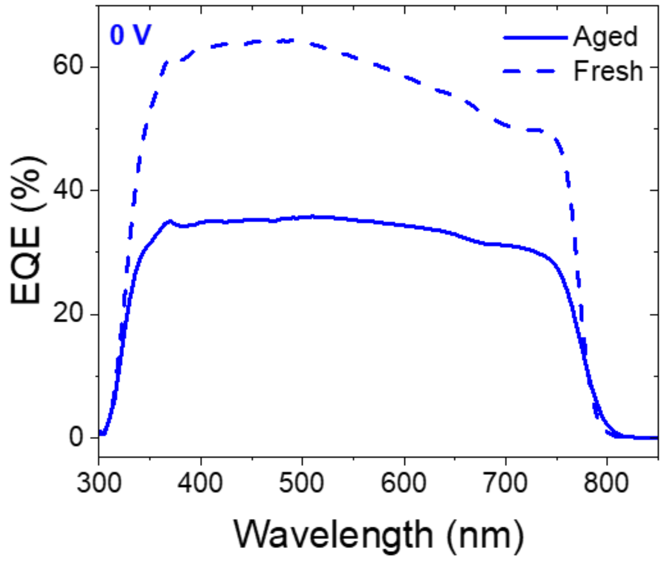

Figure S8: *Ageing for 1 year in dry air (desiccator) causes EQEs to decrease.*

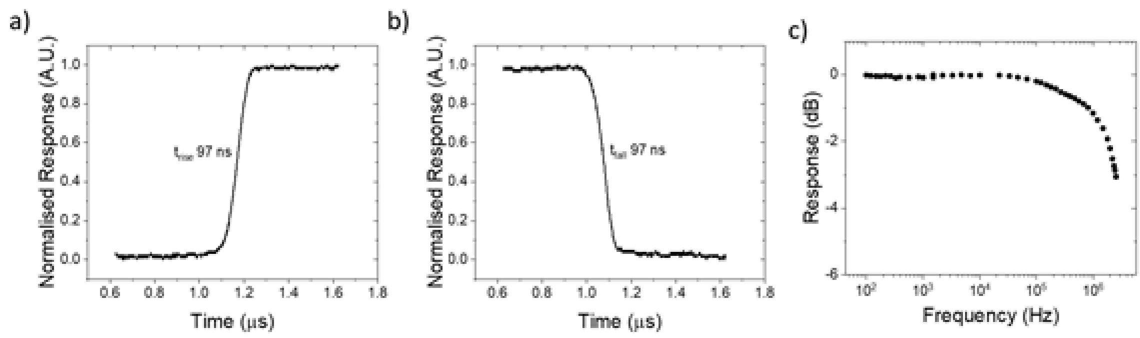

Figure S9: The response time limits of the measurement set-up, measured with an ultrafast Si photodiode (Thorlabs FDS010). *a) and b) show the rise and fall limits exceeds the*

measured perovskite photodiode response speeds. c) shows the -3 dB cut-off frequency is >1 MHz, exhibiting a wider bandwidth than the measured devices.

References

1. Fay, P. Photodetectors. in *Encyclopedia of Materials: Science and Technology* 6909–6923 (Elsevier Ltd., 2001).

2. Yang, D. & Ma, D. Development of Organic Semiconductor Photodetectors: From Mechanism to Applications. *Advanced Optical Materials* **7**, 1800522–1800522 (2019).

3. Fang, Y., Armin, A., Meredith, P. & Huang, J. Accurate characterization of next-generation thin-film photodetectors. *Nature Photonics* **13**, 1–4 (2019).

4. Dong, R. *et al.* High-Gain and Low-Driving-Voltage Photodetectors Based on Organolead Triiodide Perovskites. *Advanced Materials* **27**, 1912–1918 (2015).

5. Zhang, D. *et al.* Trapped-Electron-Induced Hole Injection in Perovskite Photodetector with Controllable Gain. *Advanced Optical Materials* **6**, 1701189 (2018).

6. Guo, F. *et al.* A nanocomposite ultraviolet photodetector based on interfacial trap-controlled charge injection. *Nature Nanotechnology* **7**, 798–802 (2012).

7. Shen, L. *et al.* A filterless, visible-blind, narrow-band, and near-infrared photodetector with a gain. *Nanoscale* **8**, 12990–12997 (2016).

8. Konstantatos, G., Clifford, J., Levina, L. & Sargent, E. H. Sensitive solution-processed visible-wavelength photodetectors. *Nature Photonics* **1**, 531–534 (2007).

9. Moehl, T. *et al.* Strong Photocurrent Amplification in Perovskite Solar Cells with a Porous TiO2 Blocking Layer under Reverse Bias. *The Journal of Physical Chemistry Letters* **5**, 3937–3936 (2014).
